# Effect of sintering parameters on physical and mechanical properties of powder injection moulded stainless steel-hydroxyapatite composite

**DOI:** 10.1371/journal.pone.0206247

**Published:** 2018-10-25

**Authors:** Mohd Ikram Ramli, Abu Bakar Sulong, Norhamidi Muhamad, Andanastuti Muchtar, Amir Arifin, Farhana Mohd Foudzi, Mohannad Saleh Hammadi Al-Furjan

**Affiliations:** 1 Department of Mechanical and Materials Engineering, Universiti Kebangsaan Malaysia, Bangi, Selangor, Malaysia; 2 Department of Mechanical Engineering, Sriwijaya University, Indralaya, Sumatera Selatan, Indonesia; 3 School of Mechanical Engineering, Hangzhou Dianzi University, PR China; University of Vigo, SPAIN

## Abstract

The combination of metallic bio-inert material, stainless-steel 316L (SS316L) and a bio-active material, hydroxyapatite (HA) can produce a composite which has superior properties for orthopaedic applications. The main objective of this study is to investigate the effects of sintering temperature and holding time on the physical and mechanical properties of the sintered part. 50wt.% SS316L and 50wt.% HA were mixed with a binder system of palm stearin (PS) and polyethylene (PE) at 61 vol.% powder loading. Rheological properties show a pseudo-plastic behaviour of the feedstock, where viscosity decreases with increasing shear rate. The feedstock was injection moulded into a tensile bar shape while thermal debinding was carried out at 320°C and 500°C. The brown parts were sintered at 1000, 1100, 1200 and 1300°C, with three different sintering times of 1, 3 and 5 hours in the furnace. It was found that the highest sintered density measured was 95.61% of the theoretical density. In addition, the highest hardness and Young’s modulus measured were 150.45 HV and 52.61 GPa respectively, which are higher than those of human bone. The lowest percentage of carbon content was 0.022wt.% given by the sample sintered at 1300°C for 1 hour. Therefore, SS316L/HA composite with good mechanical and physical properties was successfully produced through the PIM process.

## Introduction

Powder injection moulding (PIM) is an efficient process to produce small components made from ceramic or metal powders having complex geometry, high precision, excellent final properties and net-shaped products at low cost [1–3]. Such process is a combination of plastic injection moulding and conventional powder metallurgy (PM) technologies [4, 5, 6]. The PIM process consists of four main steps: mixing, injection moulding, debinding, and sintering [7–9]. In the mixing step, the powder and binder will be mixed together at a certain ratio to produce a homogenous feedstock. Next, the feedstock will undergo an injection moulding process where the feedstock will flow into the mould cavity at certain temperature to produce the green part of the desired shape [10–12]. The green part will then go through the debinding step where the binder system will be removed to obtain the brown part. Finally, the brown part is then sintered at elevated temperatures in an isotropic way to achieve the full density.

Hydroxyapatite (HA) is known as a calcium phosphate ceramic with a chemical formula of Ca_10_(PO_4_)_6_OH_2_ [13–15]. HA is a bio-active ceramic material that has excellent properties in terms of bio-compatibility, bio-affinity, osseo-conductivity while having huge chemical similarities with human bones and teeth [16–19]. HA helps in promoting bone growth by forming strong chemical bonds with natural bone after the implantation process [20, 21]. However, there is a major factor that limits the use of HA as an implant in high load bearing applications due to its poor mechanical properties [22, 23]. Poor mechanical properties of HA lead to a variety of studies have been conducted to solve this problem. Therefore, HA with good mechanical properties is required to expand its application in the bone implant application. In order to improve the mechanical properties of HA, several works have been conducted by adding a mixture of metallic element in HA and produce a metal-ceramic composite [24, 25, 26]. Such approach has been conducted Zou *et al*. [24] where he added SS316L into HA using the powder compaction process. In addition, it has been reported that Zhao *et al*. [25] coated magnesium (Mg) on HA while Thian *et al*. [26] used titanium alloy (Ti6Al4V) as a reinforcement material onto HA through the PIM process.

Generally, metals that have been widely used as implants for bones and teeth are stainless steel (SS316L), magnesium (Mg), cobalt-chromium molybdenum alloy (CoCrMo), titanium (Ti) and titanium alloy (Ti6Al4V) [25, 27, 28]. However, the ability integration rate of these metallic bio-materials to perform a direct chemical bonding between the implant material and the original bone is depending on how the metallic surface is modified [29]. By combining these metallic bio-materials with HA, it can produce a metal-ceramic composite with good physical and mechanical properties that can subsequently be effective for a long term of load-bearing applications [30]. SS316L, which is also known as an austenitic stainless steel, has been widely used as an implant material in orthopaedic applications due to its good corrosion resistance at high temperature [31]. Its advantages include availability at low cost, ease in fabrication and mechanical properties are relatable to bone mineral [32]. In addition, SS316L is a bio-compatible material and its thermal coefficients are close to that of HA ceramics [33].

Fabrication of SS316L/HA composite has been reported through many processes such as slip casting [33], dry pressing [24], hot pressing [34], coating [30, 35, 36] and spark plasma sintering [37], but none has reported on fabricating such composite using the PIM process. In this study, 50wt.% of SS316L powder is mixed with 50wt.% of HA to produce a composite through the PIM process. It is expected that by adding SS316L into HA, greater mechanical properties can be obtained compared to pure HA, hence enabling the ability to cater for load-bearing applications. The main approach of this composite is to increase the strength and toughness of HA ceramics [14].

Composites with good physical and mechanical properties can be formed with the sintering process. During such process, consolidation of the particles will occur within the product, which then turns into a strong aggregate. In addition, shrinkage and densification will also occur on the product. Gökbayrak [38] found cracks on his sintered HA implants, thus, the sintering process parameters must be optimised in order to get the desired product with good mechanical properties. Wang and Chaki [39] have reported that the critical sintering temperature for HA is 1300°C while Koseski *et al*. [40] have found that the highest density of SS316L can be achieved when it is sintered at 1350°C for 1 hour. Sintering of HA at elevated temperatures is complicated since it has the tendency to eliminate the functional group of OH in the HA matrix (also known as the dehydration process). If such functional group is removed, the HA phase will decompose into other phases known as α-tricalcium phosphate (α-TCP), β-tricalcium phosphate (β-TCP) and tetracalcium phosphate (TTCP) [41]. These changes can hinder the densification process and the mechanical properties will be deteriorated [42]. In the present work, the SS316L/HA composite is produced via the PIM method and the objective of this work is to study the effect of sintering temperature and holding time on the mechanical properties of SS316L/HA sintered part. The density, hardness, Young’s modulus and percentage of carbon content of SS316L/HA sintered parts were also investigated.

## Materials and methods

The water-atomised SS316L powder used in this research was purchased from Epson Atmix Corporation, Japan with an average particle size of 6.75μm. The HA powder was supplied from Sigma Aldrich with average particle size of 5.34μm, as measured by particle size analyser (Mastersizer 3000, Malvern Instruments, United Kingdom). The density of SS316L and HA powders were 7.92 g/cm^3^ and 3.14 g/cm^3^, respectively, which was determined by Ultrapycnometer 1000 (model UPY-21, Quantachrome Instruments, USA). The binder system that consists of 60wt.% of palm stearin (PS) and 40wt.% of polyethylene (PE) was used, based on the previous research by Omar *et al*. [43]. The melting point for each binder was determined by differential scanning calorimeter (model DSC 822E, Mettler Toledo, USA) and the degradation temperature was obtained through thermogravimetric analyser (model STA 449 F3 Jupiter, Netzsch, Germany) at a temperature of 700 °C with heating rate of 10°C/min. DSC and TGA results were then used as reference for the mixing and debinding temperatures.

Based on the critical powder volume percentage (CPVP) analysis with parameters of 25 rpm at 27 °C, the critical powder loading was 63.2vol.%. According to German and Bose [44], the optimum powder loading for the feedstock is between 2–5% below the critical powder loading. Thus, the maximum powder loading of 61vol% which is 2% from the CPVP value, was chosen for this work. Rheological properties were determined by capillary rheometer (model CFT-500D, Shimadzu, Japan) with die diameter of 1 mm at different temperatures; 150, 160, 170 and 180°C load of 100–160 kgf. The feedstock was prepared by mixing 50wt.% of SS316L and 50wt.% of HA with the binder system using the mixer (model W50EHT, Brabender, Germany). The mixing temperature was set to 150°C with speed of 25 rpm for 2 hours. Next, the feedstock was crushed into small pellets using a heavy-duty crusher machine to ease the process of injection moulding.

The SS316/HA feedstock was injected into dumbbell shape using an injection moulding machine (model DSM IM12M, Xplore, Netherland). The parameters for injection moulding process as follows: Injection temperature: 160°C, mould temperature: 100°C, injection pressure: 0.8 bar, and injection time: 7s. The injection parameters were chosen in order to get green part that is free from defects such as cracks, short shot and flashing.

The next process is the debinding process where the binder system will be removed from the green part. Based on TGA result, the debinding process is divided into two stages of temperatures; 320°C at heating rate of 3°C/min and 500°C at heating rate of 5°C/min, which are to remove PS and PE, respectively. The green parts were debound under argon atmosphere by using a split debinding furnace (model RSAT-800, RS Advanced Technology, Malaysia) and sintered at four different temperatures; 1000°C, 1100°C, 1200°C and 1300°C for 1, 3 and 5 hours, at a fixed heating rate and cooling rate of 3°C/min under air environment using a furnace (model L16, CMTS, Malaysia).

The effect of sintering temperature and holding time on the physical and mechanical properties of the sintered part were evaluated. Morphological examinations were analysed by using a scanning electron microscope (SEM) (model S-3400N, Hitachi, USA) with magnification of 5000× and the accelerating voltage of 10.0 kV after sputter coating them with gold. Energy Dispersive X-ray (EDX) analysis was done using the SEM machine to determine the elemental composition of the sintered part. The XRD analysis (model D8 Advance, Bruker, Germany) was carried out to identify the phases present in the sintered SS316L/HA part at 0.02 2theta/min step size. The density of the sintered SS316L/HA was determined based on the Archimedes principle and MPIF 42 (*Determination of Density of Compacted or Sintered Powder Metallurgy Products*) [45]. The hardness was measured by a Metallic Vickers Tester (model HVS10, MITAKA, Japan) based on ASTM E384 with load of 19.61 N and 15 seconds indentation time. A three-point bending test was conducted on all sintered parts in accordance with MPIF 41 (*Determination of Transverse Rupture Strength of Powder Metallurgy Materials*) [45] using 1 tonne universal testing machine (model 5567, Instron Division, USA) with the crosshead speed was maintained at a rate of 0.1 mm/min. Finally, the percentage of carbon content was measured by CHNS-O Element Analyser (model EA-1108, Fisons, United Kingdom) according to ASTM 1019 standard.

## Results and discussion

**Fig 1:**
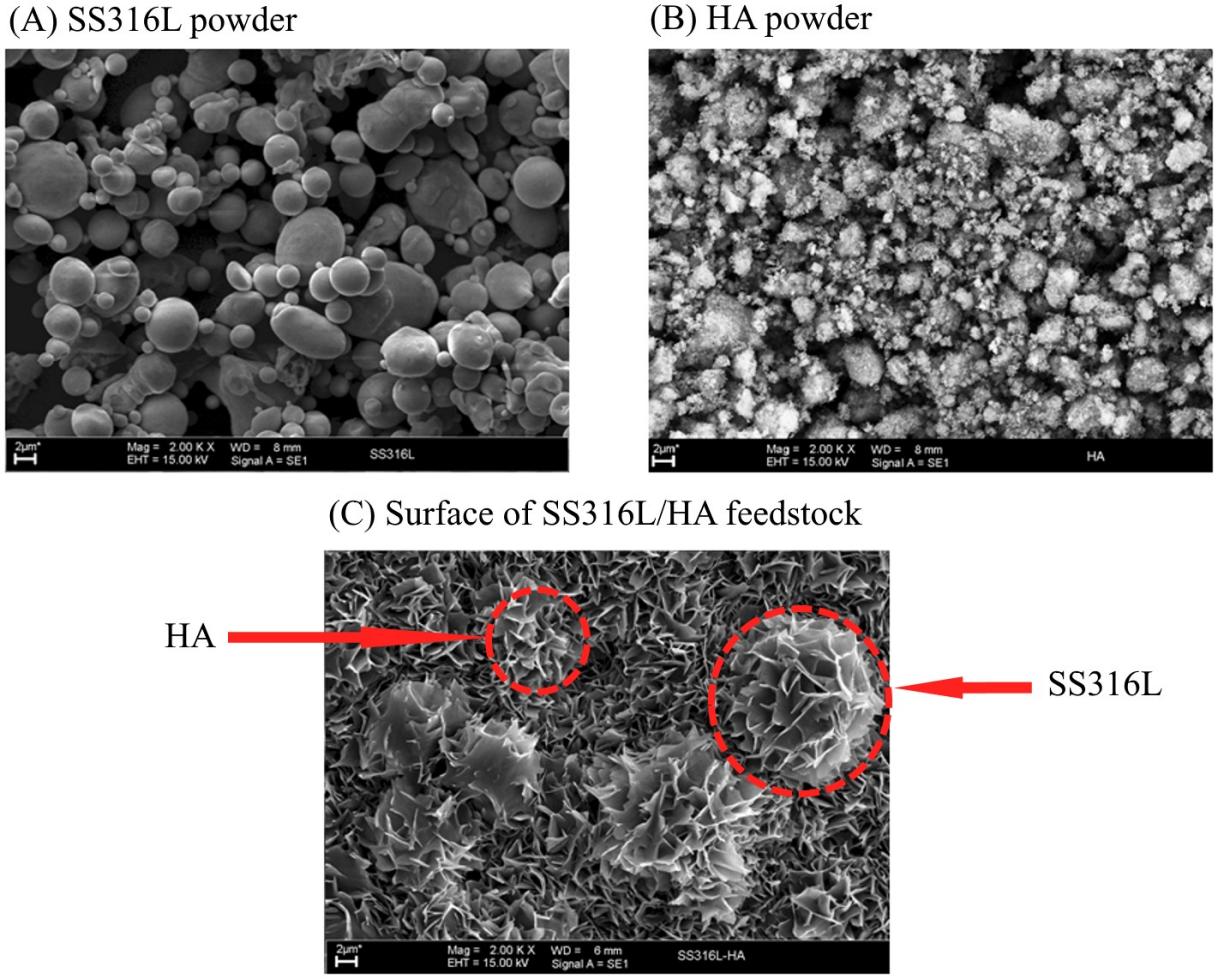
SEM micrographs of (A) SS316L powder, (B) HA powder and (C) surface of SS316L/HA feedstock.

Fig 1 shows the scanning electron microscope results of (A) SS316L powder, (B) HA powder and (C) surface of SS316L/HA composite feedstock for 61 vol.%, respectively. It was shown that the feedstock was homogeneously mixed with the HA coated the SS316L particles. Homogenous of the feedstock is very important as to avoid powder-binder separation during the injection of the feedstock into the mould [6].

**Fig 2:**
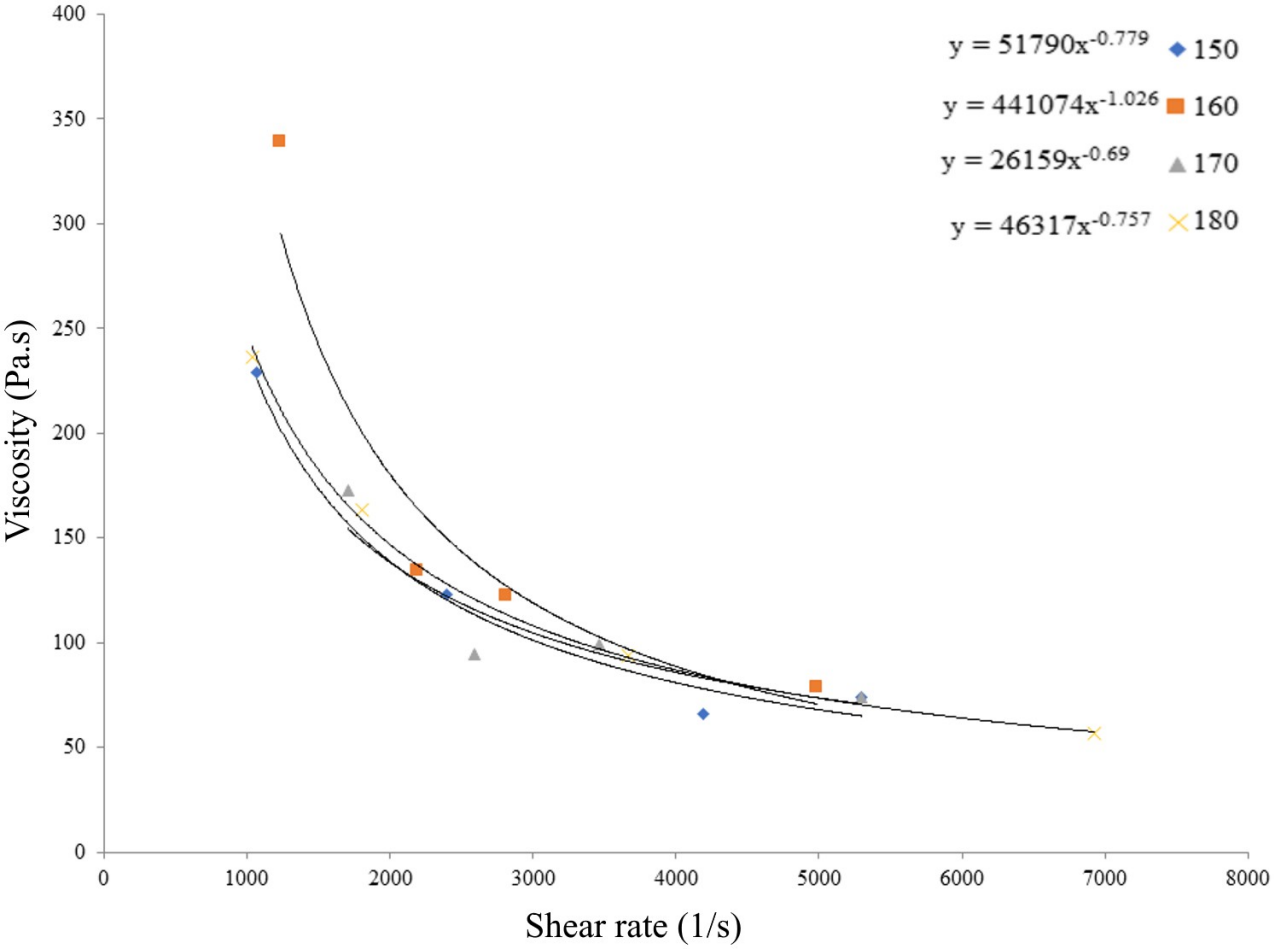
Rheological properties of SS316L/HA feedstock at powder loading of 61 vol.%.

**Fig 3:**
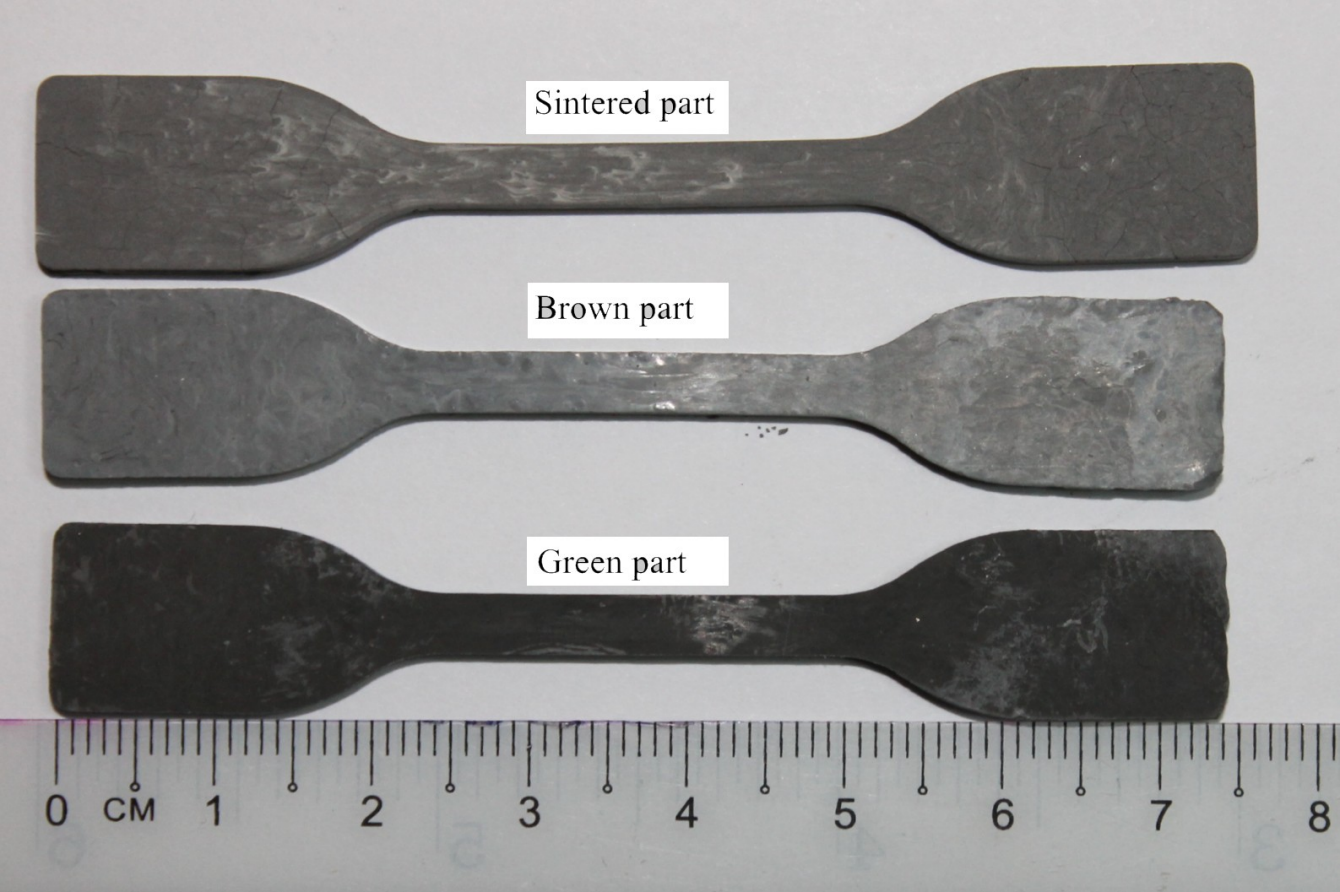
Green part, brown part and sintered part of 61 vol.% after sintered at 1000°C.

Rheological test was carried out on a feedstock of 61 vol.% powder loading to identify its flow behaviour. In PIM process, complete filling of the molten feedstock inside the mould depends on the viscosity of such feedstock with good rheological properties. Viscosity of feedstock is sensitive to temperature and shear rate [45]. The rheological properties for feedstock of 61vol% powder loading are presented in Fig 2. It was found that the viscosity decreased as the shear rate increased which indicates a pseudo-plastic flow. Huang *et al*. [46] concluded that feedstock with such flow is preferred in PIM practise where good properties of end product can be achieved. In addition, Sotomayor *et al*. [47] found that such flow produced green parts having no defects. Based on Fig 2, the viscosity and shear rate of all powder loading feedstocks were ranged between 35.01 Pa.s to 229.20 Pa.s, and 1070 s^-1^ to 11210 s^-1^, respectively. In theory, the viscosity and shear rate should be in the range of 10 Pa.s to 1000 Pa.s and 100 s^-1^ to 100000 s^-1^, respectively [44]. Therefore, powder loading of 61vol% is suitable for the injection moulding process. Fig 3 shows the green, brown and sintered parts at 1000°C for powder loading of 61 vol.%.

**Fig 4:**
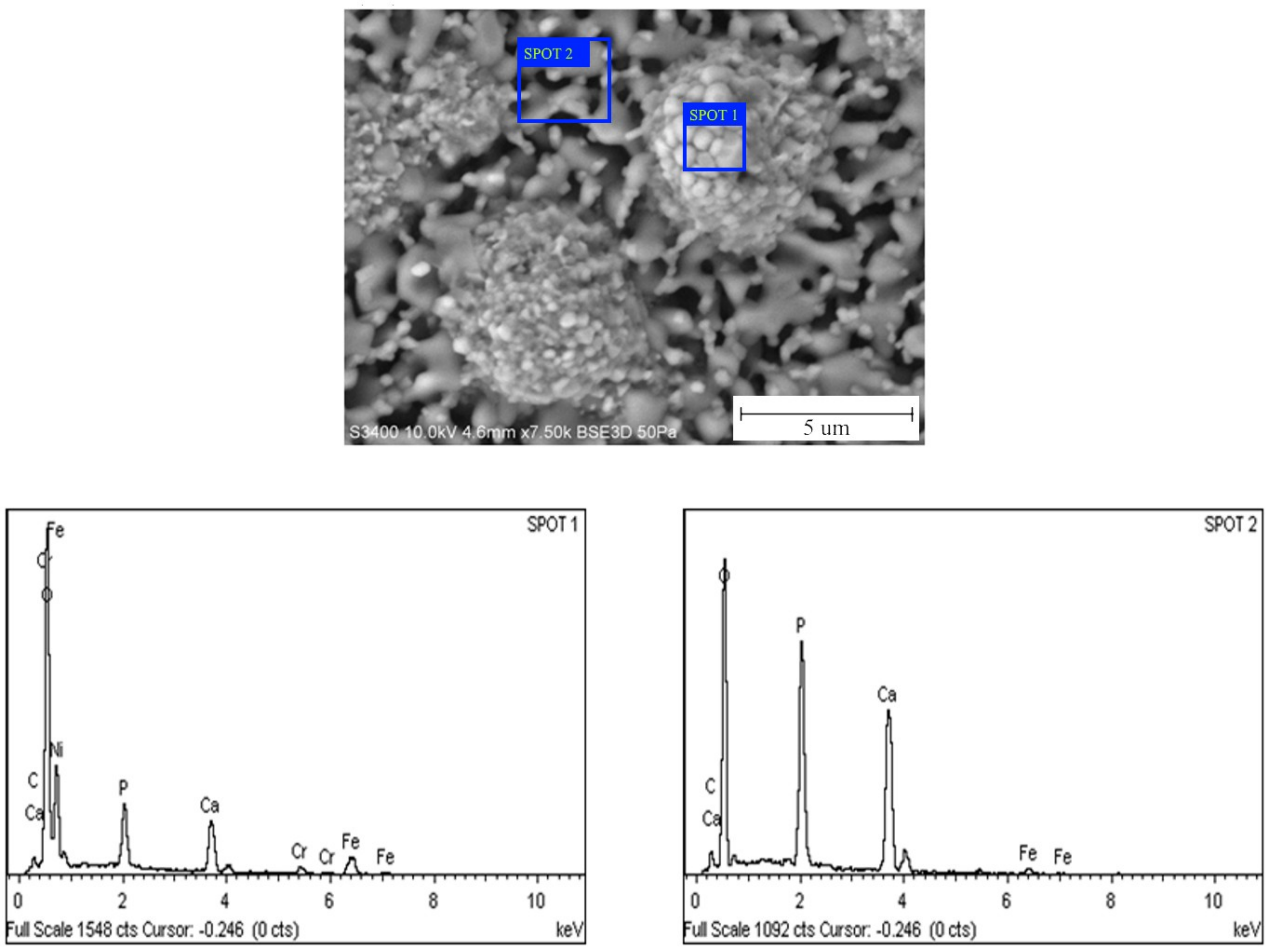
EDX analysis for 61 vol.% sintered at 1000°C for 1 hour.

**Fig 5:**
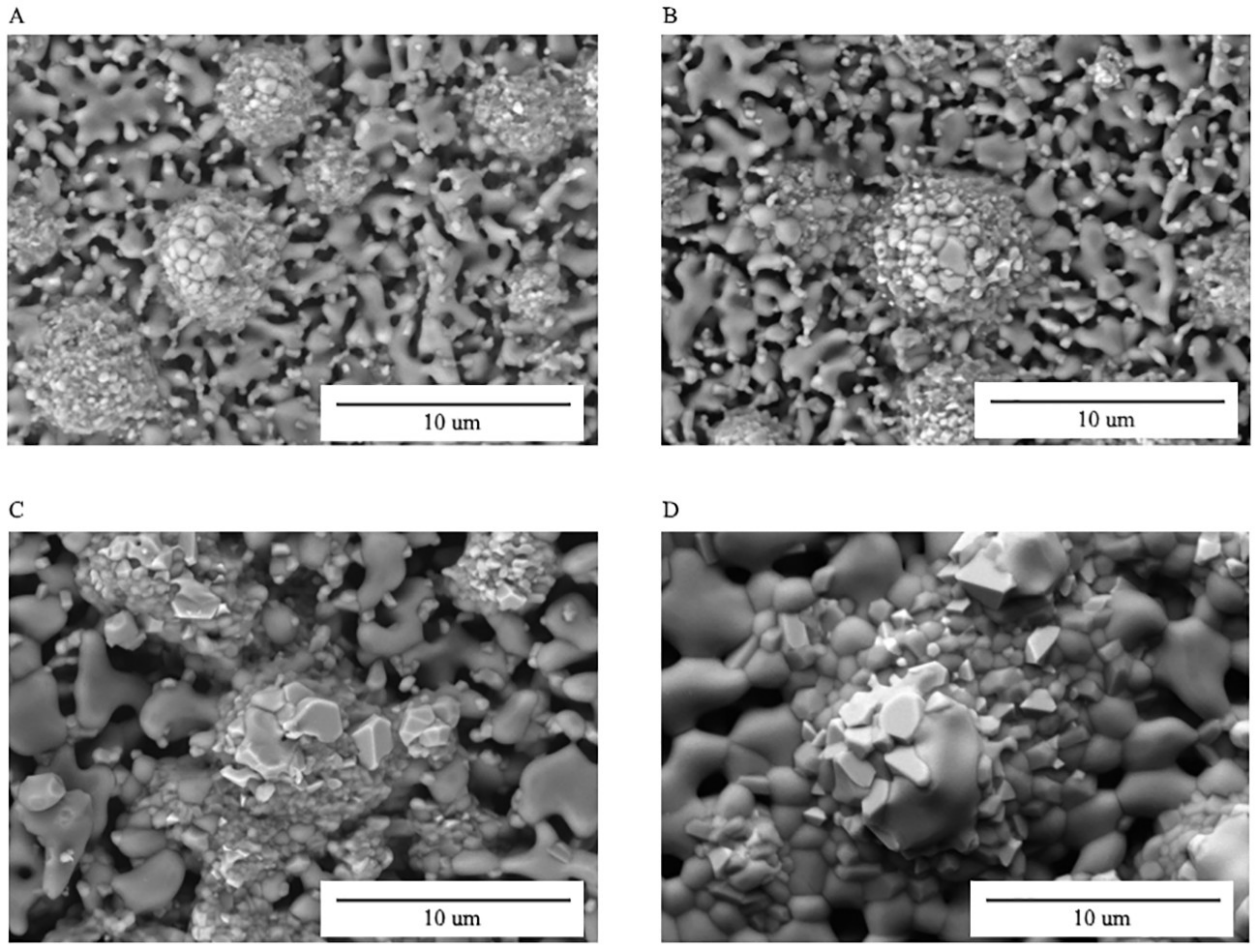
Morphology images of the sintered parts at 61 vol.% powder loading at sintering temperature of (A) 1000°C, (B) 1100°C, (C) 1200°C, and (D) 1300°C for 1 hour.

Energy dispersive X-ray (EDX) analysis was done to identify the elemental composition of the sintered part which is shown in Fig 4. Table 1 shows the weight and atomic percentage for the EDX analysis at spot 1 and 2 respectively. At spot 1, ferum (Fe) element was higher than Ca and P which is represent SS316L on the area measured. While in spot 2 area, elements of Ca and P were higher than Fe which is represent HA. Sintering temperature influences the mechanical properties of the final part in terms of its density, hardness, flexural strength and Young’s modulus [11]. Sintering was conducted on the 61 vol% brown part at different temperatures such as 1000°C, 1100°C, 1200°C and 1300°C, for 1 hour. Fig 5 shows the surface morphologies of the sintered part at (A) 1000°C, (B) 1100°C, (C) 1200°C and (D) 1300°C, respectively.

**Table 1:** Elemental composition based on Spot 1 and Spot 2 EDX analysis for sintered 61vol.%.

Element	Spot 1		Spot 2	
	Weight%	Atomic%	Weight%	Atomic%
C	1.82	41.10	2.37	4.61
O	36.22	61.10	41.90	61.06
P	6.64	5.79	17.76	13.37
Ca	11.86	7.98	31.14	18.12
Cr	4.07	2.11	0.00	0.00
Fe	34.52	16.68	6.89	2.85
Ni	4.87	2.24	0.00	0.00
Total	100		100	

It is observed that porous structure is significant at lower sintering temperature compared to higher sintering temperature. Such porous structure seems to be connected to each other, as based on Fig 5(A) and 5(B). Meanwhile, it is observed that HA and SS316L begin to diffuse with each other during the necking process. The necking process takes place between the particles during the dynamic heating stage. Such process resulted to open pore structure that remained at all sintering temperatures. Thian *et al*. [48] mentioned that such process could provide a desirable combination of high mechanical properties and good porosity. A porous structure is important because it allows body fluid circulation and absorption of protein in the bones that can improve osteo-conductivity and promotes bone growth [49]. In addition, porous structure can enhance the cell osteoblast adhesion within the pore which can promotes the formation of filopodia [50, 51]. Based on Stangl *et al*. [52], adhesion of osteoblastic cells on porous commercially pure Ti implants seem to have positive effect on growth of human feral osteoprogenitor cell line. Consequently, as the temperature increases, the grain size will also increase and result to smaller number of pores which will produce a dense sintered part. In addition, Fig 5(C) and 5(D) show that SS316L experienced significant changes in the porous structure where the grain growth is multifaceted when sintered at 1200°C and 1300°C, respectively. At the 1300°C, it is observed that the grain size of HA and SS316L increased and interconnected with each other which separated by grain boundaries. As illustrated by Song *et al*. [53], it can be concluded that the higher sintering temperature, the bigger the grain size will be.

**Fig 6:**
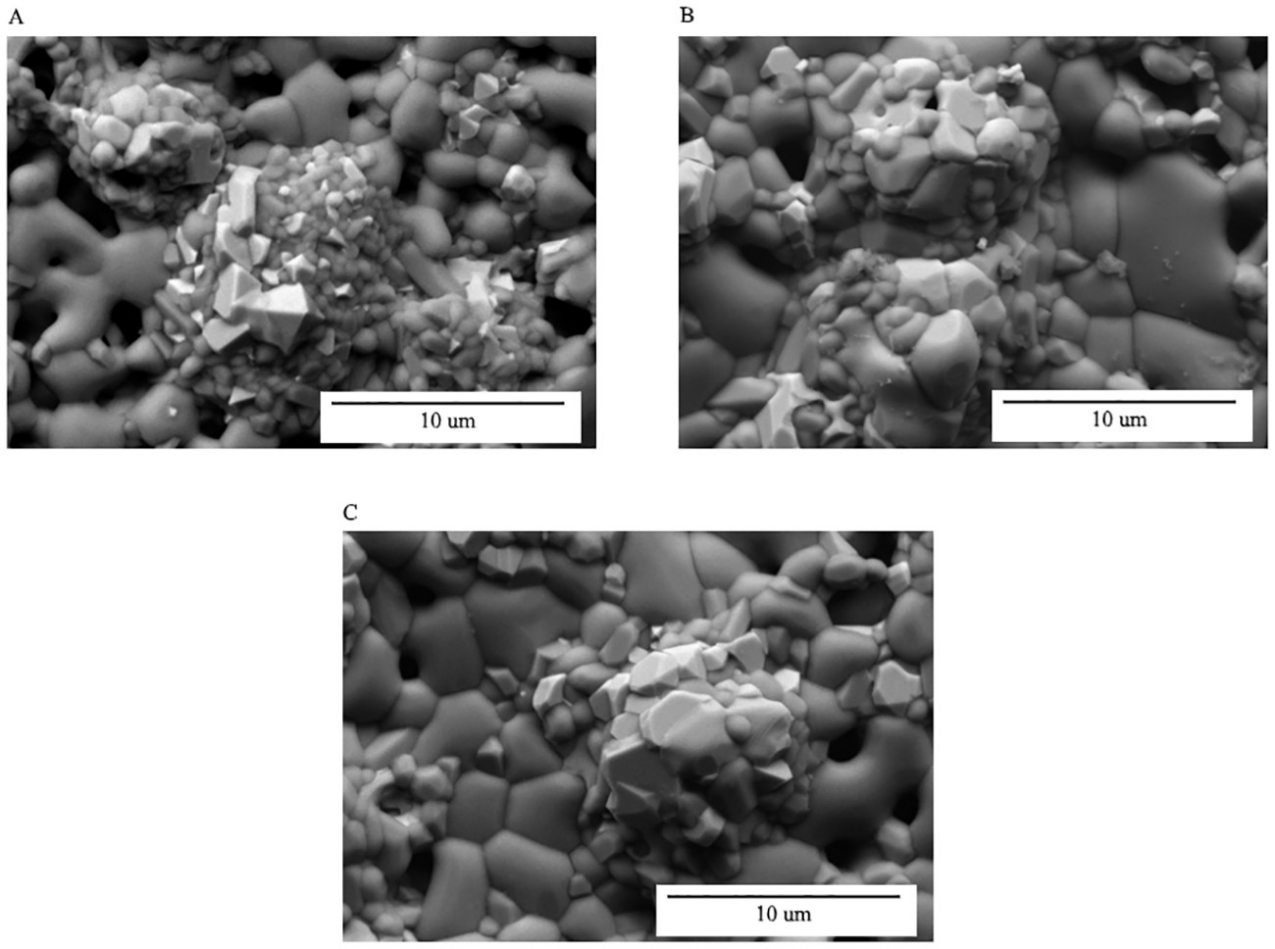
Morphology images for 61 vol.% sintered part at 1300°C for (A) 1 hour, (B) 3 hours and (C) 5 hours.

During the sintering process, the agglomeration of HA will produce empty spaces which is known as pores. Pores resulting from irregular shaped powder particles cannot be completely removed during the grain enlargement process and therefore will form close pores [54]. The air trapped in the close pores will expand and causes elongation of the sintered part. This phenomenon was reported by Lin and Hwang [55] where it was found that when the gas pressure in the closed pores is high, cracks would appear in the sintered part. Inversely, when the gas pressure is low, plastic deformation would occur and caused expansion of the sintered part. Morphological properties based on the effect of holding time of 1, 3 and 5 hours were conducted on the sintered part. Fig 6 shows the surface morphologies of 61 vol.% sintered part at 1300°C for (A) 1 hour, (B) 3 hours and (C) 5 hours, respectively. It is observed that the grain growth and pores decreased as the sintering time increased, which caused the part to be denser. However, Muralithran and Ramesh [42] stated that if the grain growth exceeds the critical grain size, the mechanical properties will be deteriorated.

**Fig 7:**
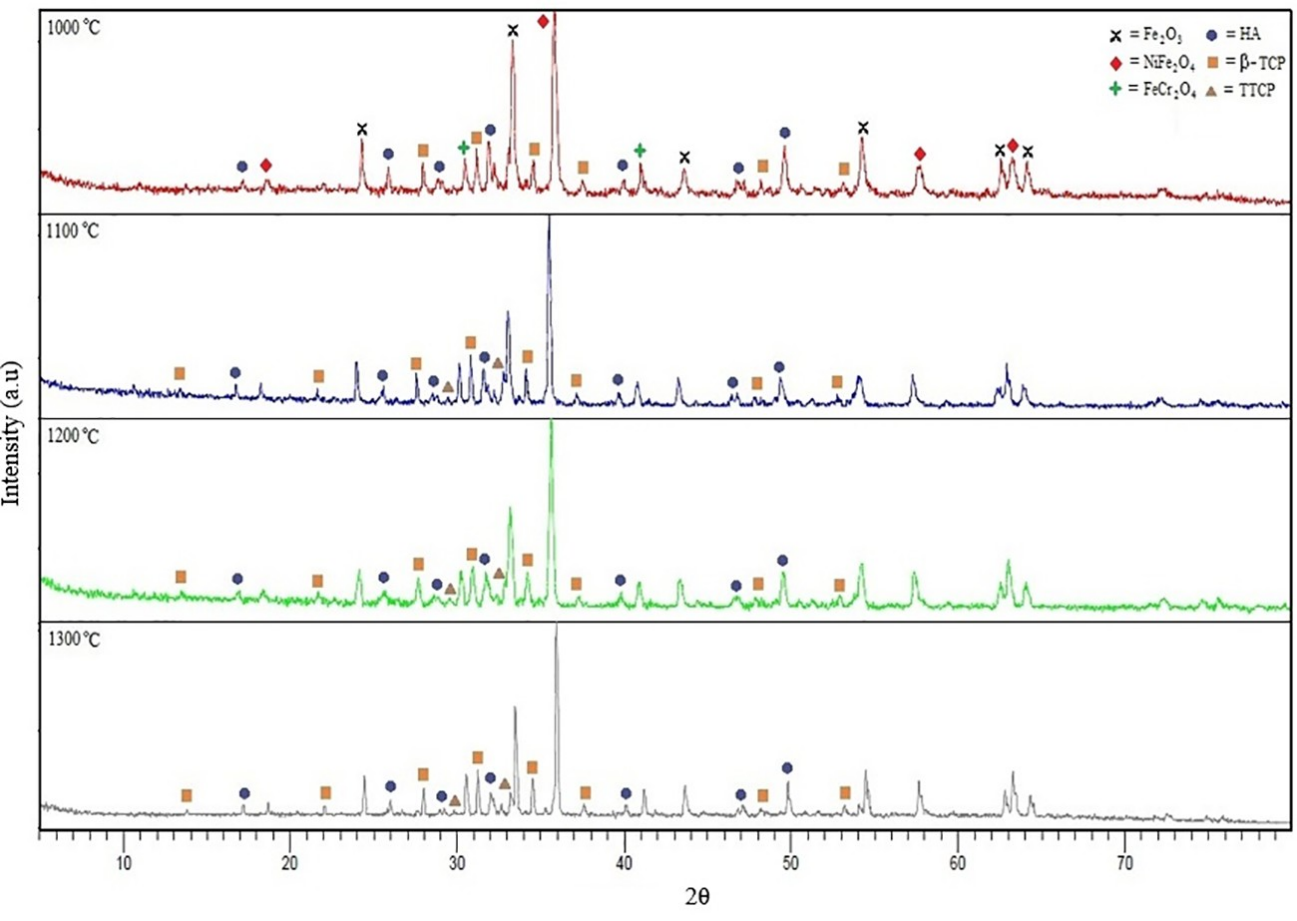
XRD plots for 61 vol.% at different sintering temperatures for 1 hour.

The phase changes in the sintered part as the sintering temperature increases was identified by XRD based on JCPDS standards such as 09–0432, 86–1585, 09–0348 and 11–0232 for HA, β-TCP, α-TCP and TCP, respectively [56]. Fig 7 shows a XRD graph for 61 vol.% sintered at different sintering temperatures for 1 hour. At 1000°C, it was observed that the HA phase has begun to decompose into β-TCP phase at ~28.2°, 31.5°, 34.1°, 37.3°, 48.5° and 53.2°. The β-TCP phase increased as the sintering temperature increased to 1300°C. In addition, decomposition of HA into the TTCP phase occurred at the sintering temperature of 1100°C which can be seen at ~30.0° and ~33.2°. Decomposition of HA to TCP and TTCP phases was due to the formation of intermediate phases and oxyapatite that formed through the loss of the OH^-^ element in the HA matrix at sintering temperature of 1100°C and above. During the sintering process, HA molecules lose the water element (H_2_O) [39]. Sintering at high temperature tends to eliminate the functional groups of HA through the dehydration process and will cause the decomposition of HA to α-TCP, β-TCP and TTCP. Such decomposition can hinder the densification process and lead to a reduction in the mechanical properties of the sintered part. Another factor that causes the decomposition of HA is the process of dehydroxylation [56]. Sridhar *et al*. [56] concluded that when HA is sinter at temperatures below its critical point, the crystal structure of HA would remain despite dehydroxylation and HA would rehydrate during the cooling process. If the sintering temperature exceeds the critical point, the dehydroxylation process will occur completely and irreversible. This causes HA structure to change and allow for decomposition to occur. The critical point is the temperature at which HA will decompose, usually in the range between ~1200°C to 1450°C [57].

**Fig 8:**
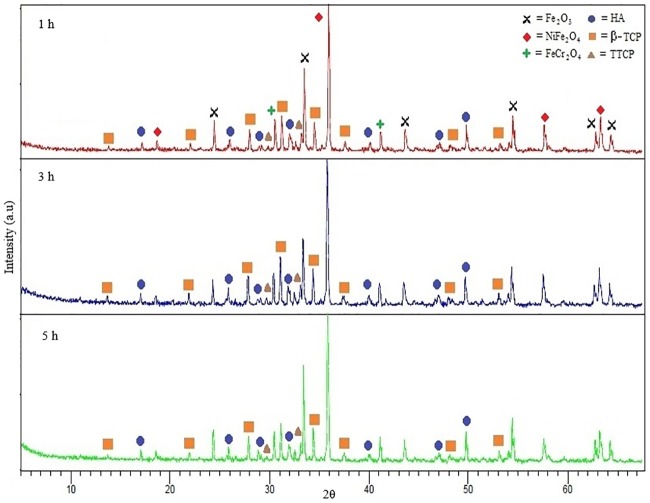
XRD plots of 61 vol.% sintered part at 1300°C for 1, 3 and 5 hours.

Comparison of JCPDS 33–0664 (hematite, Fe_2_O_3_), 34–0412 (chromite, FeCr_2_O_3_) and 54–0964 (trevorite, NiFe_2_O_4_) phases in SS316L was conducted [58]. It is found that hematite, chromite and trevorite phases were formed when SS316L is oxidised which is after the sintering process. Based on Fig 7, two peaks ~33.2° and ~35.8° were clearly observed which represent hematite and trevorite, respectively. High oxygen content caused the ions in SS316L such as chromium, nickel and iron, to react with oxygen during the sintering process. The presence of oxygen is caused by the decomposition of HA during the dehydroxylation process where OH decomposes into O^-^ and H^+^. Kijima and Tsutsumi [59] stated that H^+^ ions would form water molecules and will be eliminated during the sintering process, while the O^-^ ion reacts with metal ions to form metal oxide [16]. Fig 8 shows the XRD plots graph for 61 vol.% sintered part at 1300°C for 1, 3 and 5 hours, respectively, where no significant changes on the phases took place when the sintering time increased. This is due to the intensity peaks at all sintering times. Thian *et al*. [11] found that the sintering time will not affect the sintered part, however, the sintering temperature will influence the physical and mechanical properties of the sintered part. Cihlar and Trunec [60] had conducted a study on HA using PIM and discovered that sintering time indeed did not give any effects on the HA phase after 1–4 hours of sintering. However, the density of the sintered part was slightly increased. In addition, they also found the presence of α-TCP phase in addition to HA when sintered for 8 hours.

**Fig 9:**
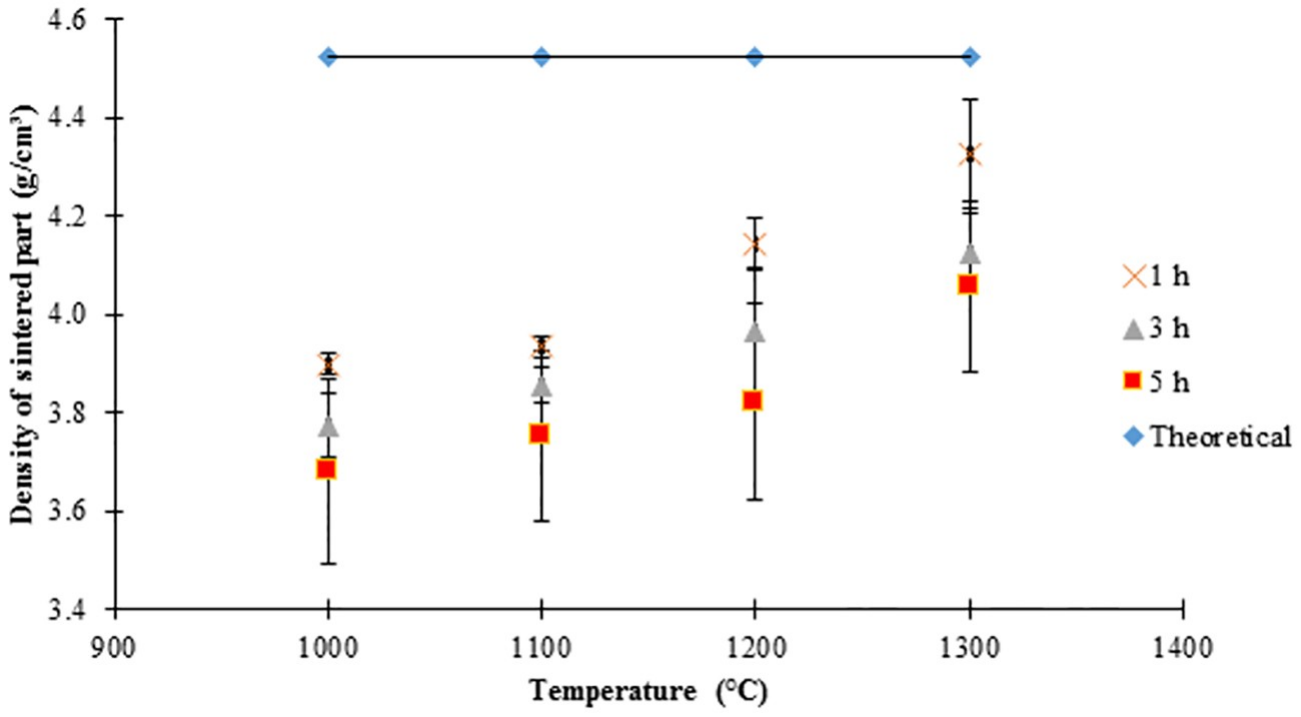
Density values of sintered part for 61 vol.% at different sintering temperatures for 1, 3 and 5 hours.

Fig 9 shows the density values of the sintered parts where the theoretical density of such part is 4.53g/cm^3^. Based on such figure, the density of sintered part increased with the increasing sintering temperature. This is due to the grain growth which resulted to sintered part to become denser although as shown by the captured images in Fig 5, the sintered parts were not fully diffused at sintering temperatures of 1000°C, 1100°C and 1200°C. Such insufficient diffusion resulted to formation of pores in the sintered part and decreased the final density. At sintering temperature of 1050°C, Lin *et al*. [61] mentioned that the grain growth process was not noticeable due to the remaining porosity that is still hindered in the sintered part which influences the final density to be lower than the theoretical density. This means that density and grain size will increase with increasing sintering temperature. According to Liu *et al*. [62], the density of sintered part did not reach 100% of the theoretical density due to the pores which indicate that air is still trapped in the sintered part. The highest sintered density for SS316L/HA composite was 95.61% (4.33g/cm^3^) after sintered at 1300°C for 1 hour. Meanwhile, the lowest density was found to be 81.24% (3.68g/cm^3^) after SS316L/HA composite was sintered at 1000°C for 5 hours. According to German and Bose [44], density of the sintered part should be within 95% of the theoretical density. As the sintering time increased from 1 hour to 5 hours, the density decreased due to the decomposition of HA to TCP and TTCP phases [42]. In addition, according to Cihlar and Trunec [60], the decrease in density is also due to the agglomeration HA powder. Meanwhile, Ji *et al*. [63] found that when the sintering temperature increased from 1200°C to 1250°C, the pores decreased while the density increased. Conversely, at sintering temperature of 1300°C, the density decreased due to the coarsening of the microstructure in the sintered part.

**Fig 10:**
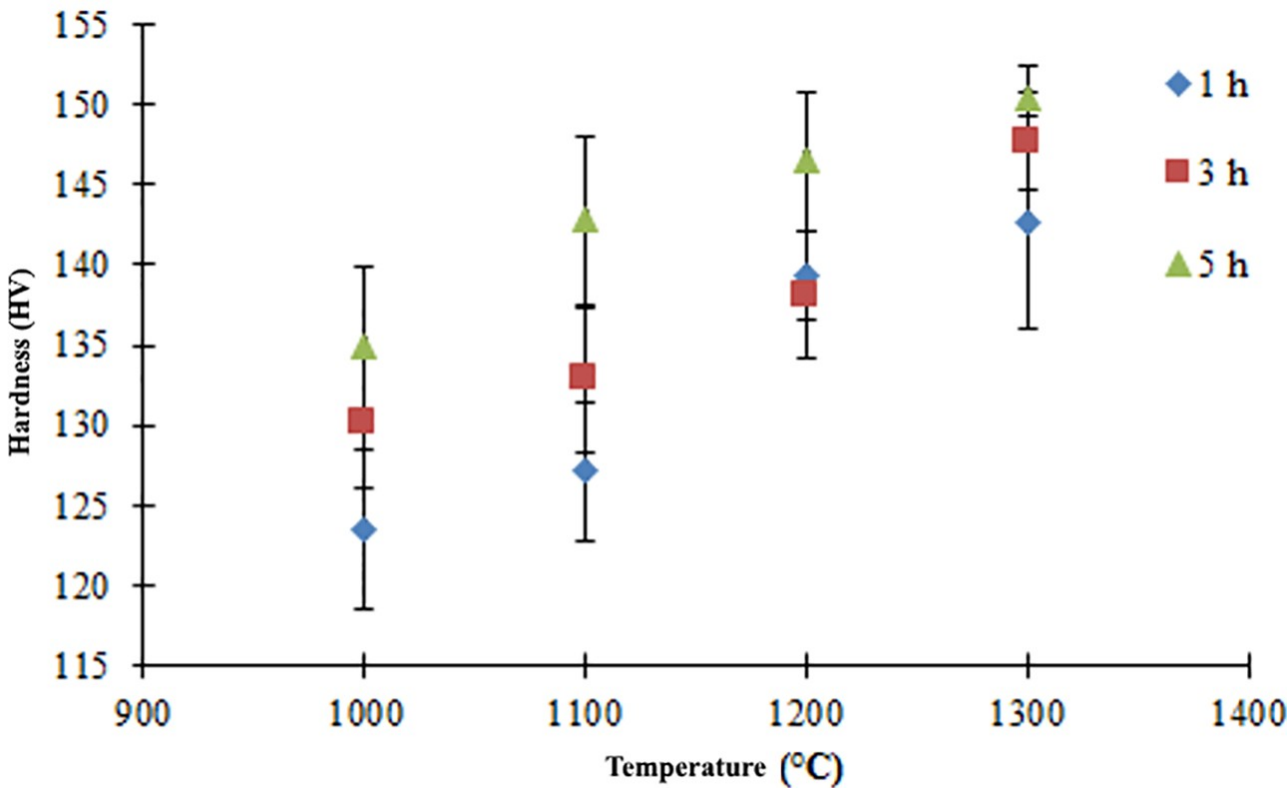
Hardness values for 61 vol.% at different sintering temperatures for 1, 3 and 5 hours.

The hardness of the final parts at 61 vol.% after sintering at different temperatures and held for 1, 3 and 5 hours, is presented in Fig 10. It is observed that the hardness increased as the sintering temperature increases. The highest and lowest values of hardness of 150.45HV and 123.51HV are obtained when sintered at 1300°C for 5 hours and 1000°C for 1 hour, respectively. By comparing with Fig 6, it is observed that the sintered part become denser as the sintering time increases. In addition, the porous area reduced which resulted to higher hardness in the sintered part. All hardness values are found to be higher compared to that of human bone where the hardness of cortical and cancellous bones are 40.38HV and 35.18HV, respectively [64].

**Fig 11:**
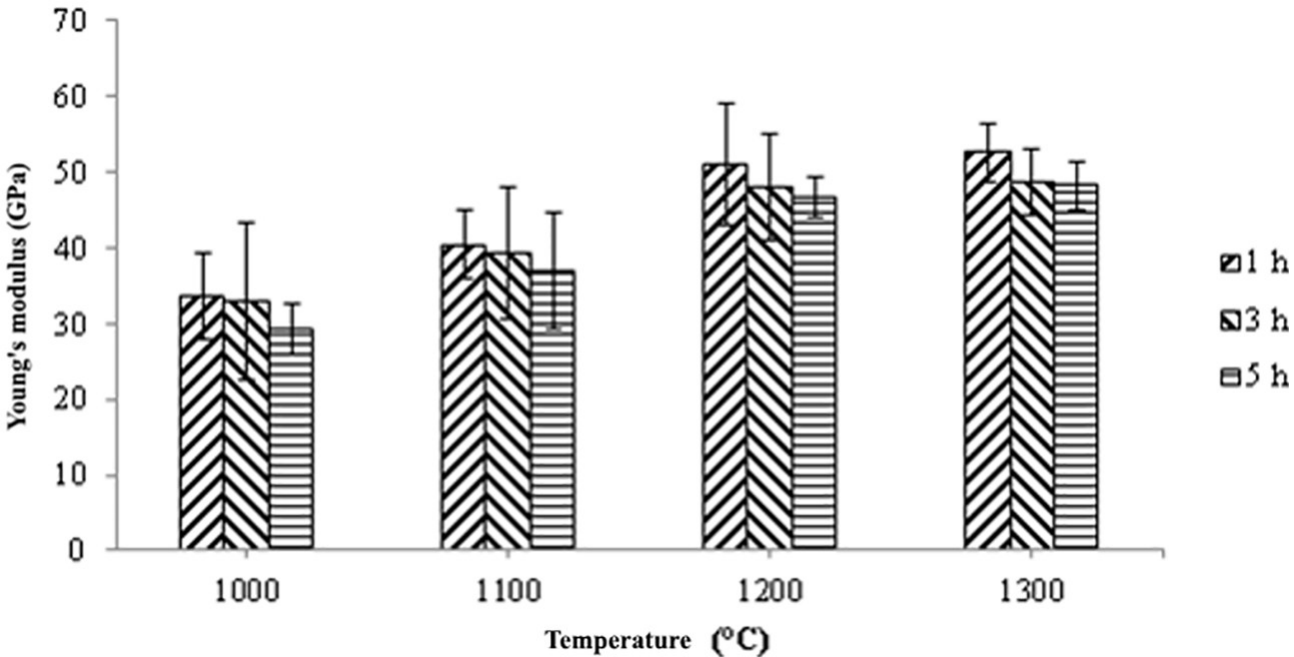
Young’s modulus values of sintered parts at 61 vol.% at different sintering temperatures for 1, 3 and 5 hours.

Fig 11 illustrates the Young’s modulus based on sintering temperature and holding time. It is observed that the Young’s modulus increased when SS316L/HA parts were sintered from 1000°C to 1300°C. In contrast, the Young’s modulus decreased as the sintering time increases. Overall, the highest range of Young’s modulus is 33.68–52.61 GPa after 1 hour of holding time at all sintering temperatures. Such values are close to the strength of human bone which is 10–30GPa [65]. According to Lee *et al*. [45], Young’s modulus for the cortical bone and cancellous bone are 18-22GPa and 0.1–0.3GPa, respectively. In addition, the obtained values are between the Young’s modulus of HA and SS316L which are ~20 GPa and ~193 GPa, respectively [66, 67]. The reason for such increment in Young’s modulus is due to the densification process that occurred between the particles. According to Callister [68], an appropriate sintering process is when the particles undergo through the necking process which leads to grain growth and less number of pores, and results to better mechanical properties of the sintered part. Prokopiev and Sevostianov [69] concluded that when the sintering temperature increased, the pore structure will change into a nearly spherical shape which results to higher strength of the sintered part (Refer Figs 5 and 6). In addition, the optimum sintering time for SS316L/HA composite was also studied. Based on Fig 11, the Young’s modulus decreases as the sintering time increased. This is due to the decomposition of HA into β-TCP and TTCP phases which may lead to poor mechanical properties of the sintered part [57, 70]. Apart from the decomposition of HA, oxidation occurred on SS316L which formed Fe_2_O_3_, NiFe_2_O_4_ and FeCr_2_O_4_. Such oxidation also contributed to the reduction of the mechanical properties of the sintered part, as reported by Muthukumaran *et al*. [71] where he found that oxidation of SS316L deteriorated the strength of the sintered part.

Carbon is one of the safest materials for the implant application due to its inert properties, biocompatible to the blood and body tissue and resistant to corrosion. On the other hand, high carbon content will decrease the mechanical properties of sintered part [48]. Based on Raza *et al*. [72], carbon content influences the performance of corrosion resistance. The carbon content percentage for 61 vol.% sintered part at 1000°C, 1100°C, 1200°C and 1300°C for 1 hour, were measured. It is found that such percentage is 0.046%, 0.025%, 0.023% and 0.022% for 1000°C, 1100°C, 1200°C and 1300°C, respectively. Therefore, it can be concluded that the carbon content percentage decreased when the sintering temperature increased. At sintering temperature of 1300°C, it is found that the lowest carbon content percentage is 0.022%. According to ASTM A240, the carbon content percentage for SS316L is 0.030wt.%, which is higher than the obtained values after sintered at 1100°C, 1200°C and 1300°C. Overall, 1300°C was found to be the most suitable sintering temperature for SS316L/HA composite.

## Conclusions

The rheological properties of the SS316L/HA composite show a pseudo-plastic behaviour and suitable for the powder injection moulding process. The effect of different sintering temperatures and holding times on SS316L/HA composite was successfully investigated. It was found that when SS316L/HA composite was sintered at 1000°C, the particles were not well sintered which resulted to more pores that were initially formed after the thermal debinding process. Such pores decreased with increasing sintering temperatures. Grain growth process caused the sintered part to become denser as the holding time increased from 1 hour to 5 hours. The HA phase began to change to β-TCP phase when sintered at 1000°C while changed to TTCP phase at 1100°C through the decomposition process. Meanwhile, no changes were observed in the intensity when the holding time increased. The density increased with the increasing sintering temperature where the highest density value was 4.33g/cm^3^ after 1 hour of sintering at 1300°C. In addition, as HA decomposed into TCP and TTCP phases, the density was found to be decreased. It was found that the Young’s modulus of the sintered parts was higher than that of human bones. As the sintering temperature increases, hardness and Young’s modulus will also increase due to the densification process. However, the Young’s modulus was found to be decreased as the sintering time increases. Finally, sintering at 1300°C gave the lowest weight percentage of carbon content of 0.022wt.%.
